# Lessons and Applications of Omics Research in Diabetes Epidemiology

**DOI:** 10.1007/s11892-024-01533-7

**Published:** 2024-01-31

**Authors:** Gechang Yu, Henry C. H. Tam, Chuiguo Huang, Mai Shi, Cadmon K. P. Lim, Juliana C. N. Chan, Ronald C. W. Ma

**Affiliations:** 1 Department of Medicine and Therapeutics, The Chinese University of Hong Kong, Hong Kong, HKSAR China; 2 Chinese University of Hong Kong- Shanghai Jiao Tong University Joint Research Centre in Diabetes Genomics and Precision Medicine, Hong Kong Institute of Diabetes and Obesity, The Chinese University of Hong Kong, Hong Kong, HKSAR China; 3 Laboratory for Molecular Epidemiology in Diabetes, Li Ka Shing Institute of Health Sciences, The Chinese University of Hong Kong, Hong Kong, HKSAR China

**Keywords:** Diabetes, Epidemiology, Biomarkers, Genetics, Epigenetics, Biobanking, Phenotype, Precision medicine

## Abstract

**Purpose of Review:**

Recent advances in genomic technology and molecular techniques have greatly facilitated the identification of disease biomarkers, advanced understanding of pathogenesis of different common diseases, and heralded the dawn of precision medicine. Much of these advances in the area of diabetes have been made possible through deep phenotyping of epidemiological cohorts, and analysis of the different omics data in relation to detailed clinical information. In this review, we aim to provide an overview on how omics research could be incorporated into the design of current and future epidemiological studies.

**Recent Findings:**

We provide an up-to-date review of the current understanding in the area of genetic, epigenetic, proteomic and metabolomic markers for diabetes and related outcomes, including polygenic risk scores. We have drawn on key examples from the literature, as well as our own experience of conducting omics research using the Hong Kong Diabetes Register and Hong Kong Diabetes Biobank, as well as other cohorts, to illustrate the potential of omics research in diabetes. Recent studies highlight the opportunity, as well as potential benefit, to incorporate molecular profiling in the design and set-up of diabetes epidemiology studies, which can also advance understanding on the heterogeneity of diabetes.

**Summary:**

Learnings from these examples should facilitate other researchers to consider incorporating research on omics technologies into their work to advance the field and our understanding of diabetes and its related co-morbidities. Insights from these studies would be important for future development of precision medicine in diabetes.

## Introduction

There has been a marked increase in the prevalence of diabetes with 420 million people affected globally [1]. Traditional population- and family-based epidemiological studies have provided important insights on distribution of disease in time, place and person as well as trends over time. These data have generated hypotheses regarding aetiologies and mechanisms. Recent advances in genotyping and the study of genetics and other molecular markers together with the availability of different high-throughput platforms used to investigate different omics layers have ushered an exciting era in omics research. When these technologies are applied to well-characterized epidemiological cohorts, they can provide deeper insights on aetiology, disease pathways, prognosis and causation to improve the precision of diagnosis and classification for personalized treatment [2]. Public health workers and practising physicians with interests in epidemiology and precision medicine are in a prime position to set up new cohorts or leverage existing cohorts to discover diagnostic and risk stratification tools as well as drug targets aimed at preventing and improving clinical outcomes. In this review article, we provided a brief overview on how omics research could be incorporated into the design of current and future epidemiological studies. Throughout the review, we drew on examples from the literature, as well as our own experience of conducting omics research using the Hong Kong Diabetes Register and Hong Kong Diabetes Biobank, as well as other cohorts, to illustrate the value of adding omics research into epidemiological studies. Learnings from these examples should facilitate other researchers to consider incorporating research on omics technologies into their work to advance the field and our understanding of diabetes and its related co-morbidities.

## Preparation for Omics Studies and Sample Considerations

Whilst there is a wide spectrum of omics that can be considered, ranging from information on the genome to epigenome, and the transcriptome, proteome, metabolome and others, the needs of the project need to be balanced against the costs and time required for collecting the required biospecimens and subsequent processing costs. Therefore, the best time to plan for omics studies is definitely before embarking on a new epidemiological study, so that the main focus of the project can be addressed, whilst also trying to “future proof” the cohort by considering any potentially necessary samples for future research. That said, it is possible to add the collection of specific samples after a project has commenced. For example, it is not uncommon for genetic data to be added during the follow-up phases of a longitudinal cohort. Whilst it is true that the DNA sequence cannot be changed and hence obtaining DNA at a later stage for genotyping or sequencing would not give rise to results that are different to results that would be generated if the DNA samples were collected at baseline, other non-coding changes in DNA, such as methylation or other epigenetic changes, including leukocyte telomere length (LTL), do change with time and environmental exposure, and hence would differ at follow-up compared to baseline, hence highlighting the additional benefit of having considered this upfront, and the potential benefit of serial collection of DNA to search for changes in these epigenetic marks. Table 1 summarizes some of the different types of omics research, and some general comments regarding the sample types and key considerations.

**Table 1:**
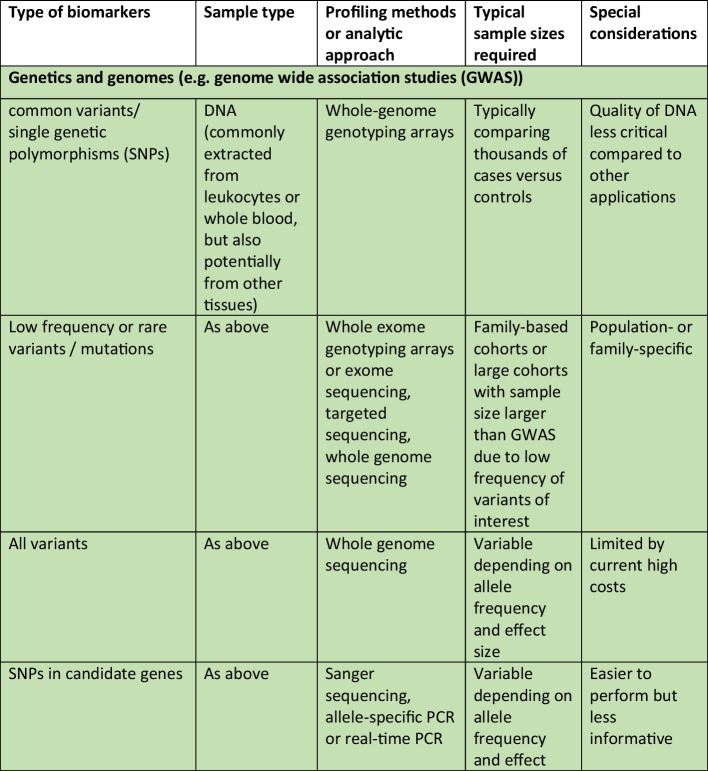

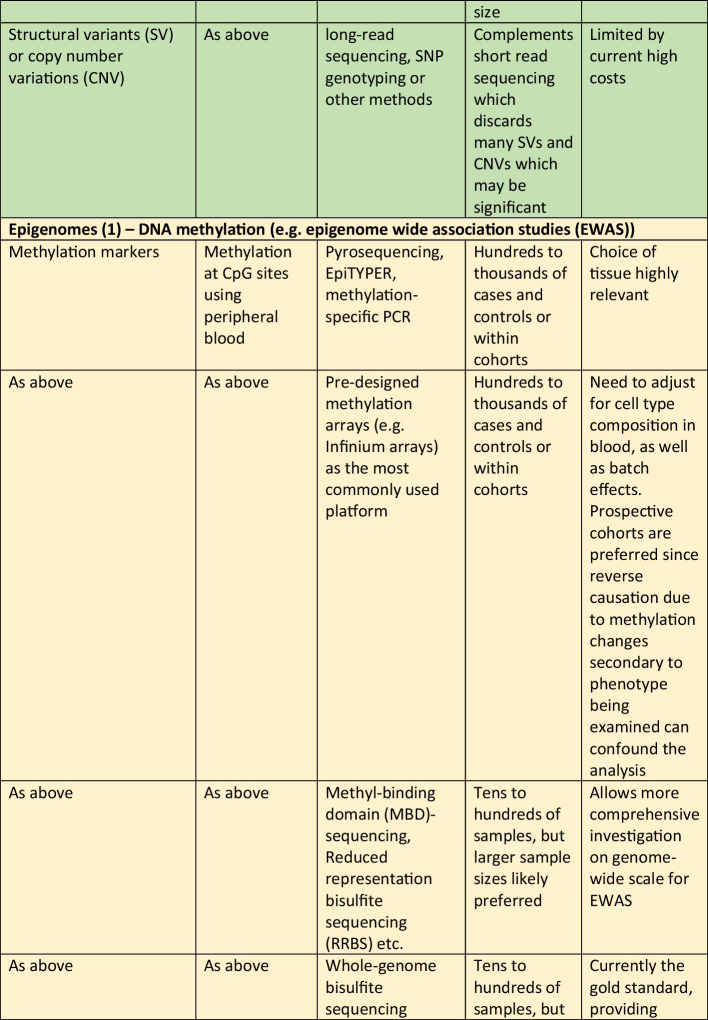

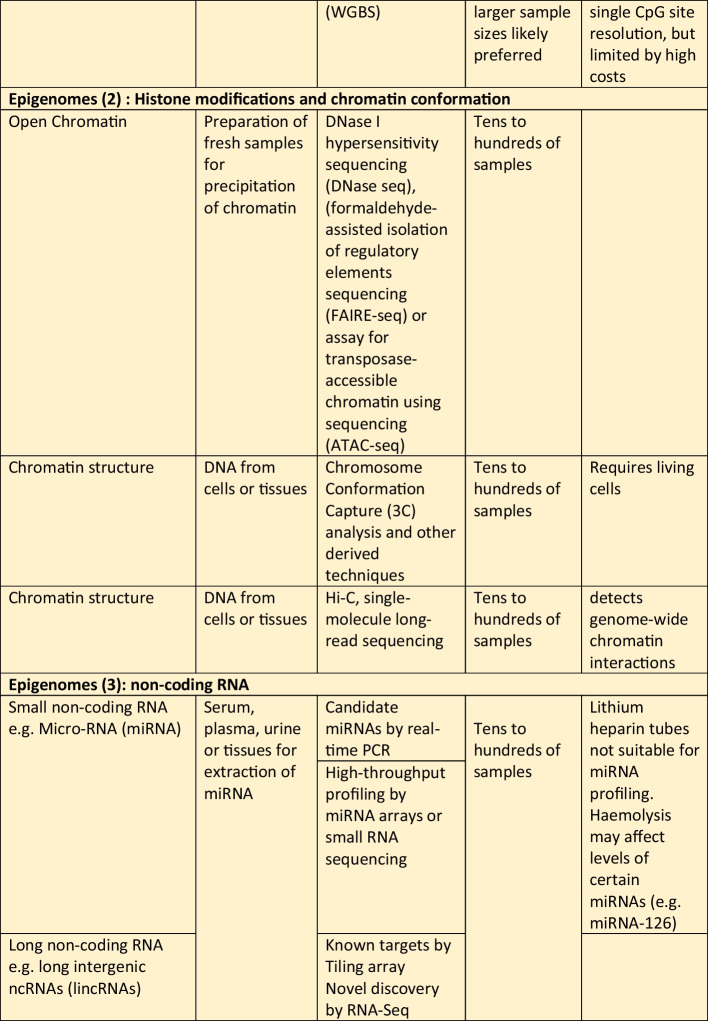

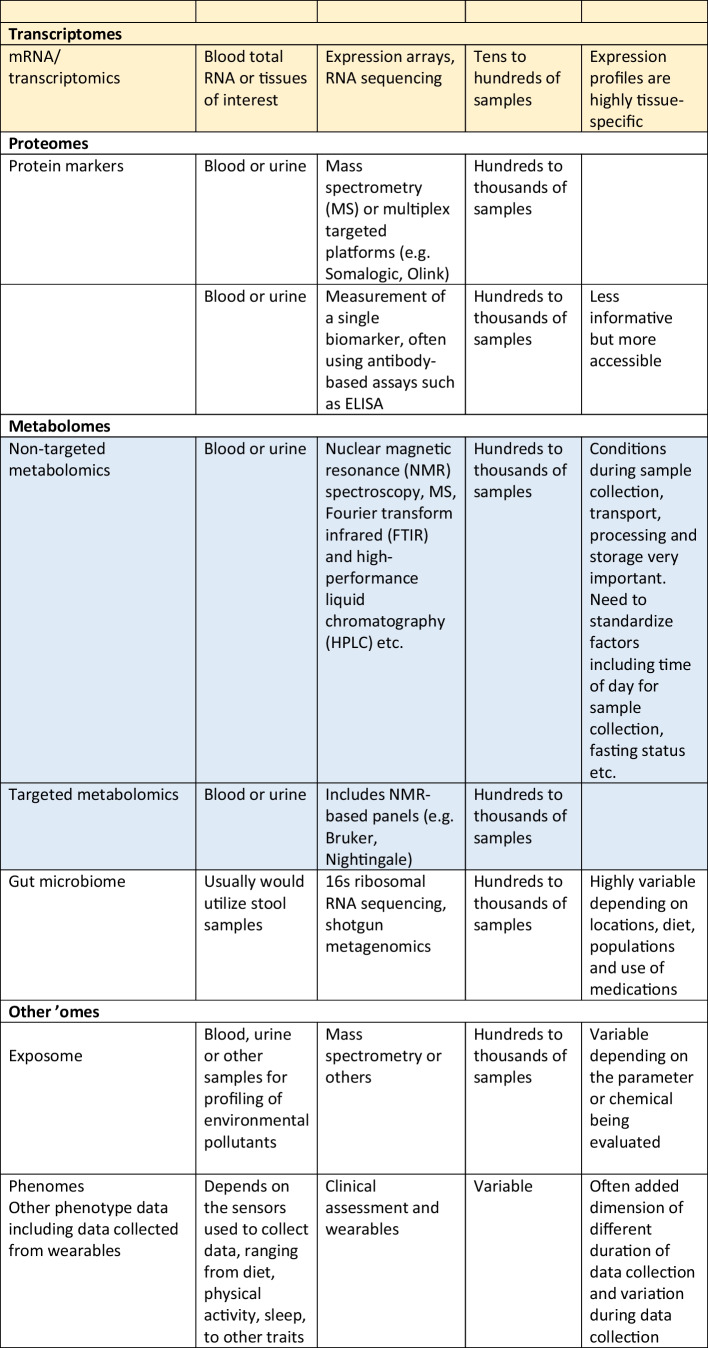
Examples of the different types of omics research, the types of samples required and special considerations in the collection of samples and biobanking procedures in epidemiological studies. Abbreviations in the table: *CpG* region of DNA where a cytosine occurs next to a guanine nucleotide, *DNAase* deoxyribonuclease, *NMR* nuclear magnetic resonance, *PCR* polymerase chain reaction, *RRBS* reduced representation bisulfite sequencing, *SNP* single nucleotide polymorphism, *WGBS* whole genome bisulfite sequencing

Another key issue that warrants some discussion is pre-analytic considerations. In addition to collecting the right types of specimens for subsequent omics profiling, it is important to bear in mind the different characteristics or pitfalls of analysis using the different platforms. Whilst genetic variations represent stable and permanent changes in the DNA sequence, methylation changes are subject to aging, effects of environment and treatment. On the other hand, metabolomic markers are more dynamic and unstable, and subject to metabolic perturbations as well as degradation. Whilst most protein markers are relatively stable, peptide hormones such as insulin are easily degraded. Hence, sample collection, transport and storage need to take into account the need to preserve the most labile or unstable analytes, and the adoption of best practice for sample collection and biobanking can enhance the success of subsequent omics profiling. Minimal sample handling, ensuring minimal sample transport and ensuring low temperature during transit and temperature monitoring during transit before storage at − 80 °C are some of the necessary steps to ensure samples are suitable for metabolomics profiling [3]. Further details on biobanking are available from more specialized reviews on this topic, and the white papers from the UK Biobank represent another good source of information [4]. We should highlight that whilst these omics technologies can be applied to different sample types, we have focused our discussion on the analysis of samples that are most likely to be collected in the setting of epidemiological investigations, such as blood samples.

## Genetic Studies, GWAS and Polygenic Risk Scores

Family studies have revealed notable genetic components for diabetes and diabetic complications [5–8]. Genetic studies of diabetes and its complications however underwent several phases as the molecular technology evolved [9]. As one of the earliest efforts, linkage analysis had limited discoveries of genetic loci with robust large effects. Candidate gene studies, another approach commonly adopted, were prone to false positives and confounders. Early genome-wide association studies (GWAS) provided an alternative, high-throughput solution, but suffered from insufficient sample sizes to identify loci with modest effect sizes, which in fact account for the majority of the genetic component underlying complex traits [10]. With the rapid development of sequencing technologies, establishment of large biobanks and collaborative meta-analyses aggregating a large number of diabetes cohorts over the last decade, major breakthroughs have been achieved in the understanding of the genomics and genetics related to diabetes. Numerous studies conducted in diverse populations have greatly advanced our understanding on the genomic architecture and genetic heritability of complex diseases including diabetes. Similar studies have been undertaken in the Hong Kong Diabetes Register (HKDR) [11, 12] and Hong Kong Family Diabetes Study (HKFDS) [13, 14] using these approaches, ranging from linkage analyses [15], candidate gene studies [16], GWAS [17] and meta-analyses [18, 19], to more recent sequencing efforts.

Diabetes is classically divided into an earlier-onset autoimmune form (type 1 diabetes; T1D) and a later-onset non-autoimmune form (type 2 diabetes; T2D), which account for the two main types of diabetes. Genetic exploration of T1D has mainly focused on the human leukocyte antigen (HLA) region, which presents the strongest association with T1D [20–22]. In addition, more than 60 loci outside of the HLA region have also been linked to T1D, including common variants revealed by GWAS in genes involved in immune regulation such as *IL2RA* and *CTLA4* [23–25], as well as rare variants underscored by targeted sequencing of known loci, e.g. *PTPN22* [26]. Although sequencing studies are known to complement GWAS by providing a more comprehensive characterization of genetic variation in a locus, there are currently limited large-scale sequencing efforts carried out in individuals with T1D.

The fast-growing epidemic of T2D and its devastating complications have spurred greater efforts in studying the genetics of T2D [27]. Earlier work in the genetic exploration of T2D has been extensively discussed elsewhere [28, 29]. To date, the largest GWAS on T2D is a multi-ancestry meta-analysis of more than 2.5 million individuals including over 0.4 million T2D cases [30]. This study identified 1289 distinct association signals mapped to 611 loci reaching genome-wide significance. Index genetic variants in the most recently identified disease loci tend to be predominantly common (minor allele frequency [MAF] over 5% in at least one ancestry group) and had small effect sizes (odds-ratios [OR] < 1.1). Apart from the expansion of known T2D loci, another contribution of this study was the detection of ancestry-correlated heterogeneity enriched at the T2D associations, which provided explanation to the varied allelic effects among populations of different ancestry groups.

Protein-coding variants or variants with low frequency have long been considered one of the explanations for the missing heritability, whereby common variants cannot fully account for the heritability for complex diseases like T2D [31]. An exome-array association study in over 80,000 T2D cases and 370,000 controls from five population groups reported 40 distinct coding-variant associations across 38 loci at study-wide significance, among which 16 (40%) association signals were located beyond known T2D loci [32]. Population-specific analyses and trans-ethnic meta-analyses revealed similar associations at the reported loci in European-descent populations and the overall study population, except for a variant in *PAX4* that was unique in East Asians [27]. Interestingly, although the study had increased power and sufficient coverage to capture low-frequency variants, 35 (87.5%) of the 40 independent coding-variant associations were common (MAF > 5%) and with modest effects (ORs 1.02–1.36). The remaining five signals also did not exert stronger effects than expected on the susceptibility to T2D, with ORs ranging from 1.09 to 1.29. Notably, fine-mapping analysis of the associated variants in the context of regional linkage disequilibrium (LD) by large-scale GWAS data only confirmed the causality of 16 (40%) signals, where one-third of the top coding-variant associations were likely to be driven by local non-coding variations nearby. Such “false leads” could result in an inappropriate inference on the disease etiology. Therefore, coding variants prioritized in genetic association studies, though functional, might not necessarily be causal. Careful investigation is required to correctly interpret the findings and the underlying biological significance.

As sequencing technologies become more high throughput and the costs continue to drop, whole-exome and whole genome sequencing (WES/WGS) have been increasingly applied to the study of the genetics of complex diseases [33, 34]. So far, the largest sequencing study in diabetes reported exome sequencing analyses of over 20,000 individuals with T2D and ~ 24,000 non-diabetic controls [33]. Gene-level association analysis of rare variants (MAF < 0.5%) identified 4 genes at exome-wide significance: *MC4R*, *PAM*, *SLC30A8* and *UBE2NL*. Of note, the association of *SLC30A8* with T2D was driven by 90 missense variants, where the reduced protein activity was linked to decreased T2D risk. However, a comparison of rare and common variant associations in subjects with both sequencing and genotyping data suggested that sequencing had limited power in detecting single-variant associations when compared with imputed array-based genotype data. In addition, even the strongest gene-level signals for rare variants resulted from sequencing data only explained 25% of the heritability of the strongest single-variant signals for common variants, arguing against conventional speculation that rare variants might have a large contribution to T2D heritability. Power analysis in the study further emphasized the need for large sample size in sequencing studies, where the authors estimated more than one million samples would be required for rare-variant signals in validated T2D therapeutic targets to reach exome-wide significance. These results highlight that sequencing study and array-based GWAS should be complementary in understanding the genetics of complex diseases like diabetes, with the former identifying informative alleles and the latter for locus discovery and fine-mapping. With the anticipated drop in sequencing costs, the integration of WES/WGS with available GWAS data in well-characterized cohorts will remain the main approach for identifying causal genes for diabetes in the near future. Nevertheless, it is also important to note that for complex diseases with partial penetrance, the concept of multicausality and the interaction of genetic predisposition with exposures over the life course are important considerations in these analyses [35, 36], and the availability of deep phenotyping, as well as the application of these principles in data interpretation, are important to improve understanding based on the genetic results generated.

Similar strategies have been applied to identify the genetic basis of diabetic complications and related outcomes, though the progress of these efforts lags significantly behind the studies on the genetics of T2D, mainly due to the limited sample size of studies so far conducted, and the heterogeneity of definitions being used for the outcomes of interests, such as diabetic kidney disease [37, 38], in part due to natural history of disease and modification by interventions. A detailed discussion on the genetics of diabetic complications is beyond the scope of this review, and is covered in more detail elsewhere [39, 40].

### Polygenic Risk Scores for Diabetes

Recent advances in GWAS for diabetes have helped drive progress in the prediction of individual genetic susceptibility to diabetes. One such development is the polygenic risk score (PRS), which aggregates the effects of genetic variants into one summary score. There have been increasing studies highlighting the utility of PRS not only for risk stratification, but also for predicting diabetes progression, clinical outcomes, treatment response and disease subtyping [41–43].

For T1D, the majority of genetic risk is accounted for by the HLA class II (DR-DP loci), HLA class I and other non-HLA loci including *INS* and *PTNP22* [44]. Previous studies have demonstrated the inclusion of PRS into models with clinical parameters could improve T1D risk prediction [45–47] and aid discrimination of T1D from T2D [48–50], or from the general population [51]. Oram and colleagues [48] developed a 30-SNP PRS for T1D (denoted as *GRS-1*) that comprised HLA and non-HLA variants, which was shown to accurately distinguish between T1D and T2D (area under the curve [AUC] = 0.88) and predict progression to insulin deficiency (AUC = 0.87) in young adults with diabetes. The second version of Oram’s T1D GRS (known as *GRS-2*) was extended to 67 HLA and non-HLA variants that comprehensively integrate DR-DQ risk, HLA interactions and additional non-HLA SNPs [49]. T1D *GRS-2* significantly improved the discrimination (AUC = 0.93) of T1D from T2D and from controls. However, currently most T1D PRSs were developed and tested only in populations of European ancestry, which could restrict its generalization and transferability in non-European populations [43, 52]. Of note, non-European populations have ancestry-specific variants, different frequencies of specific alleles and haplotypes, and peculiar subtypes like fulminant T1D reported in China, Japan and India [53•].

In parallel, T2D PRSs have also been found to be of use for risk stratification in the general population, or help with prediction of disease progression in T2D patients [54]. A study by Mahajan and colleagues [55], utilizing data from UK Biobank, found that individuals in the top 2.5% of the PRS distribution were at 9.4-fold increased risk compared with the bottom 2.5%. Meanwhile, in a Chinese cohort of patients with T2D from the HKDR, a T2D PRS consisting of 123 T2D-associated SNPs predicted rapid progression to insulin therapy [56]. Further, increasing GWAS from non-European populations [57–59], large-scale trans-ancestry meta GWAS [54, 60] and novel cross-population/trans-ancestry PRS methods [61] has facilitated the transferability of T2D risk prediction in diverse populations. For instance, based on a Bayesian polygenic modeling method, PRS-CSx [62], Ge and colleagues [61] constructed a trans-ancestry T2D PRS by integrating T2D GWAS from European, African and East Asian populations and demonstrated that the top 2% of the trans-ancestry PRS distribution can identify individuals of European, African, Hispanic/Latino and East Asian ancestry with an approximately 2.5–4.5-fold of increase in T2D risk, which corresponds to a similar increased risk of T2D for first degree relatives of someone with T2D.

T2D is increasingly recognized as a collage of varied pathophysiological states with heterogeneous etiology [63]. In a similar vein, T2D PRS, as a unitary summary score, may not fully capture the complexity of the heritable risk. According to the “palette” model [64••], a series of underlying pathophysiological processes contribute towards the development of T2D. Therefore, increasing attention has been drawn to partition PRS by genetic clustering or decomposition, in order to achieve more personalized risk prediction. Udler and colleagues have decomposed the genetic associations across diabetes-related biomarkers and identified five T2D genetic clusters using a “soft” clustering method [65], which was further extended into ten clusters in their recent work [66]. These clusters/partitioned PRSs showed distinct patterns of associations with metabolic traits and clinical outcomes related to T2D [65–67], suggesting the great potential of process-specific PRS to provide mechanistic insights into the heterogeneity of T2D and advance precision medicine in diabetes. Besides, fine-mapping, functional annotation and partition of T2D loci by integrating the transcriptome and epigenome information could help develop mechanism-driven tissue-specific PRS. Miguel-Escalada and colleague [68] constructed an islet-specific T2D PRS using islet enhancer hub variants involved in islet gene regulation and insulin secretion and showed its potential in delineating T2D risk profiles from the novel perspective of tissue-specific epigenomic mechanisms.

However, one should exercise caution when interpreting the results of risk stratification based on PRS because PRS alone may not perform so well on its own, but can be improved when PRS are considered in combination with existing risk factors [69]. Therefore, the clinical utility of PRS for diabetes should be assessed combining other clinical risk factors, such as BMI (body mass index), GADA (glutamic acid decarboxylase antibodies), and fasting glucose and insulin. Besides, diabetes is the product of the combination and interaction of genetic (G) and environmental (E) factors. It still remains unclear whether and how GxE interactions influence the effect of PRS. In other words, the effect of the T2D PRS would depend on the level of the environmental risk factors like lifestyle, socioeconomic status and environment pollutants. Merino and colleague [70] explored if T2D PRS and diet quality have a synergistic effect on the risk of T2D and found only independent associations between the two factors and incident T2D. Therefore, evaluating the interactions between T2D PRS and environmental factors in diverse populations could help improve the transferability of PRS and unravel the clinical heterogeneity of diabetes.

Whilst many epidemiological cohorts have contributed to the identification of genetic factors for diabetes, analyses increasingly require collaborative analyses across many cohorts involving participants from different ethnic populations taking into consideration the clinical contexts, e.g. ecological anthropology and healthcare environment. Nevertheless, genetic studies have highlighted the potential of epidemiological cohorts to contribute towards biological understanding of common diseases. Discoveries from WGS/WES and GWAS will have different utilities and are complementary. PRS from GWAS can be used for prognostication and patient segmentation, and can be used as companion diagnostics whilst WGS/WES will provide drug targets for modifying disease pathways. Applying these 2 technologies to prospective cohorts with detailed phenotyping along with interventions provide an important strategy for precision medicine.

## Epigenomics, miRNA and Other Epigenetic Biomarkers

Along with genetics, the regulation of gene expression can be achieved through epigenetic modifications without altering the DNA sequence [71]. There are multiple mechanisms involved in the epigenome including non-coding RNA regulation, DNA methylation and histone modifications. These mechanisms are critical in controlling gene expression, cell development and differentiation. As environmental exposures and external factors can modify the epigenome, dysregulation of the epigenome is thought to provide a picture integrating environmental as well as germ-line genetic variation which may contribute to the development of different chronic diseases, including diabetes and related co-morbidities [72, 73]. Furthermore, epigenetic changes have been implicated to mediate glycaemic or metabolic memory, whereby previous exposure to hyperglycaemia may lead to sustained effects on gene expression and increased risk of diabetes complications [74•, 75].

MicroRNAs (miRNAs) are a class of non-coding and single-stranded RNAs with lengths of 18–25 nucleotides [76, 77]. MiRNAs play an important role in the post-transcriptional regulation of gene expression by epigenetic modulation [78]. Capable of RNA interference, miRNAs can bind with target messenger RNAs (mRNAs) to inhibit translation or induce mRNA degradation [79]. Since miRNAs are involved in an extensive range of biological processes including cell development, differentiation, proliferation and apoptosis, they have been proposed as potential disease biomarkers [76, 80•]. With high stability and resistance to degradation in human biofluids, circulating miRNAs have emerged as promising biomarkers for diabetes [76] and diabetic complications [80•, 81].

In a recent sequencing-based analysis of islet miRNAs, human pancreatic islet samples were used for RNA sequencing, small-RNA sequencing and genotyping. Eighty-four miRNAs were found to be highly heritable, and mainly regulated by trans-acting genetic effects. In addition, the study identified 14 T2D-related miRNAs including miR-21-5p and miR-187-3p [82]. High-throughput quantitative real-time PCR (qPCR) is another commonly used approach in miRNA profiling. OpenArray and Dynamic Array are high-throughput qPCR platforms that require small reaction volumes (nanoliters) for each reaction. They have been applied to validate a miRNA signature generated by small-RNA sequencing in diabetic retinopathy [83].

From the HKDR cohort, screening of miRNA markers for liver cancer in T2D patients was conducted using serum-extracted RNA. Applying microarray for discovery and qPCR validation, miR-122-5p and miR-455-3p were identified to be potential biomarkers to predict the development of liver cancer [84]. Circulating miRNAs could also be used to predict the development of T2D. Gestational diabetes mellitus (GDM) is a common pregnancy complication and is associated with sevenfold increased risk of T2D in later life [85]. Using postpartum plasma, an observational study explored the potential of circulating miRNAs to predict the future development of T2D. OpenArray and qPCR were applied for discovery and validation, with miR-369-3p identified to be significant after validation and multiple comparisons. It is suggested that the measurement of this miRNA could improve the subsequent prediction of T2D in women with GDM [86].

DNA methylation is another important epigenetic modification, occurring mainly in the CpG islands [87]. DNA methyltransferases (DNMTs) mediate DNA methylation by covalently adding a methyl group to the 5′ position of the cytosine residue, leading to transcriptional silencing [74•]. In an early epigenome-wide association study (EWAS) using methylation arrays in the London Life Sciences Prospective Population (LOLIPOP) study, 5 methylation markers identified from methylation profiling of peripheral blood were found to be associated with incident T2D, including a site in the *TXNIP* gene, a methylation locus which has consistently been replicated since [88]. Interestingly, a methylation score constructed from the top 5 loci was strongly associated with incident diabetes across cohorts, independent of established risk factors [88]. Findings from such EWAS efforts complement findings from epigenomic profiling of diabetes-related tissues such as adipose tissue, which found differential methylation in some novel loci, in addition to established T2D-related genes, such as PPARG, KCNQ1 and TCF7L2 [89].

The Diabetes Control and Complications Trial (DCCT) and the long-term follow-up in the Epidemiology of Diabetes Interventions and Complications (EDIC) Study were landmark studies which highlighted the persistent impact of a period of suboptimal glucose control, subsequently named “metabolic memory”, on the progression of microvascular outcomes in people with T1D [74•, 90]. Profiling of DNA methylation in leukocyte and monocyte DNA from participants who experienced metabolic memory and microvascular complications, compared to participants in the intensive control arm who were free of diabetes complications, identified differential methylation at a number of key loci, including hypomethylation at cg19693031 in the 3′-untranslated region (3′-UTR) of *TXNIP* [91]. This is particularly interesting given the established role of *TXNIP* in hyperglycaemia [88]. A study conducted in Native Americans with T2D identified methylation loci associated with baseline renal function and subsequent decline in renal function [92]. A study in the HKDR, with methylation profiling of 1271 patients with T2D, identified 40 CpG sites significantly associated with baseline eGFR, and 8 CpG sites associated with decline in eGFR. A prediction model was developed to estimate eGFR slope using methylation data, which was replicated in the Native American population. The model was also useful for improving prediction of end-stage renal failure among people with diabetes [93]. Interesting, the CpG sites were near genes enriched for functional roles in kidney disease, and several of the CpG sites identified showed association with renal fibrosis [93].

Histone modifications refer to the post-translational covalent addition of functional groups to histone proteins. These include histone H3 lysine 4 trimethylation (H3K4me^3^) and H3/H4 lysine acetylation (K_ac_) associated with active gene expression, as well as H3K9me^2/3^, H3K27me^3^ and/or DNA methylation that are usually associated with repressed gene expression [74•]. In participants from DCCT/EDIC who experienced metabolic memory and went on to develop microvascular complications had enrichment of the active chromatin mark H3K9ac, which was associated with the mean HbA1c during follow-up. Furthermore, the hyperacetylated promoters were enriched for genes involved in inflammatory pathways, highlighting the potential role of epigenetics in metabolic memory and diabetes complications [94].

Leukocyte telomere shortening is a biomarker of biological aging, and may represent another type of epigenetic marker in diabetes [95]. In the HKDR, relative leukocyte telomere length (rLTL) was found to be inversely associated with the risk of incident diabetic cardiovascular and renal complications [96, 97].

Summary of some applications of epigenetic biomarkers from the above studies are shown in Table 2.

**Table 2:** Examples of epigenetic biomarker applications in diabetes

Epigenetic biomarker	Study subjects	Sample types	Detection Methods	Findings	References
miRNA	Human individual	Pancreatic islet	RNA-sequencing, small-RNA sequencing and genotyping	14 T2D-related miRNAs	[82]
miRNA	Individuals with or without diabetes	Serum	Next-generation sequencing and qPCR technologies (ViiA 7, OpenArray, Dynamic Array)	High-throughput qPCR can be applied for quantitative assessment of miRNA signature	[83]
miRNA	T2D patients from HKDR cohort	Serum	Microarray and qPCR	miR-122-5p and miR-455-3p are potential biomarkers for development of liver cancer in T2D	[84]
miRNA	Women with GDM	Postpartum plasma	OpenArray and qPCR	miR-369-3p is a potential biomarker to predict T2D in women with GDM	[86]
DNA methylation	Incident T2D patients in LOLIPOP study	Peripheral blood	Methylation arrays, next-generation sequencing and pyrosequencing	5 methylation markers including a site in TXNIP gene are associated with incident T2D	[88]
DNA methylation	Individuals with or without diabetes complications	Genomic DNA of whole blood and blood monocytes	Methylation arrays and pyrosequencing	Differential methylation at a number of loci	[91]
DNA methylation	Individuals with T2D and with chronic kidney disease	Peripheral blood	Methylation arrays	Methylation loci associated with baseline and decline in renal function	[92]
DNA methylation	T2D patients from HKDR cohort	Genomic DNA of whole blood	Methylation arrays	CpG sites associated with baseline eGFR and eGFR slope respectively	[93]
Histone modification	DCCT/EDIC participants	Peripheral blood	Chromatin immunoprecipitation (ChIP)—qPCR	Association between HbA1c and H3K9ac, and potential role of epigenetics in metabolic memory	[94]
Relative leukocyte telomere length (rLTL)	T2D patients from HKDR cohort	DNA from whole blood	qPCR for rLTL measurements	rLTL is inversely associated with incident diabetic cardiovascular and renal complications	[96, 97]

These studies demonstrate the potential of epigenetic markers to improve diagnosis and outcome prediction for precision medicine in diabetes [2, 98]. Another area where epigenetic biomarkers are of particular interest is in the developmental origins of diabetes and the potential impact of maternal hyperglycaemia and nutrition on epigenetic changes [99]. A detailed discussion of this is beyond the scope of this review, and readers who are interested in this area are referred to review articles which provide more details [99–101].

## Applications of Proteomics in Diabetes

Proteomics, an analytical discipline dedicated to exploring the dynamic fluctuations in protein composition, expression and post-translational modifications, has been instrumental in elucidating the pathophysiological mechanisms underlying metabolic disorders such as diabetes mellitus [102]. Affinity-based assays, which commonly employ either monoclonal or polyclonal antibodies tethered to a reporter molecule via luminescence, fluorescence, radioactivity or enzymatic activity in enzyme-linked immunosorbent assays (ELISA), remain the most commonly used approach for protein identification and quantification [103]. Nevertheless, these assays are inherently constrained by their selectivity for a single analyte of interest, and therefore cannot provide an unbiased measure of all proteins in the sample. Moreover, the low abundance of most serum proteins poses significant challenges to their detectability via traditional assays [104]. Mass spectrometry (MS) has emerged as a robust alternative, effectively employed in the identification and quantification of proteins such as galectin-1 and apolipoprotein A-1, both of which exhibit altered expression profiles in diabetic individuals [105]. MS methodologies can be executed in a targeted or non-targeted modality, affording either high specificity in protein identification or simultaneous quantification of multiple analytes, even those present in low concentrations. Nonetheless, MS entails a laborious and temporally extensive workflow, necessitating the depletion of high-abundance plasma proteins, mechanical protein separation, trypsin digestion and subsequent verification via immunoassays or other confirmatory protocols [106, 107].

Recent advancements in proteomic profiling technologies have appreciably augmented the efficacy and scope of detectable circulating proteins. Affinity-based techniques that incorporate antibody multiplexing or innovative affinity reagents have substantially broadened the quantitative capabilities of these assays. High-throughput methodologies such as nucleic acid affinity reagents (aptamers) or nucleotide-labeled antibodies have become increasingly prevalent. The SomaLogic platform employs aptamers, capitalizing on the structural versatility of oligonucleotides to specifically bind protein epitopes, thereby facilitating protein quantification [108]. Conversely, the Olink platform employs nucleic acid–labeled antibodies, enabling the utilization of polymerase chain reaction (PCR) technology for protein amplification, detection and quantification [108]. Nonetheless, the specificity of binding remains an inherent limitation, which can be mitigated through corroborative studies employing traditional immunoassays, MS and integrative genomic analyses [109, 110].

A Swedish study utilized nucleic acid–labeled antibodies and proximity extension assay to identify seven circulating proteins associated with the homeostatic model assessment of insulin resistance (HOMA-IR). These included the novel association of cathepsin D, as well as previously reported proteins such as leptin, renin, IL-1ra (interleukin-1 receptor antagonist), hepatocyte growth factor, FABP4 (FA-binding protein 4) and tPA (tissue-type plasminogen activator). However, the associations of IL-1ra and tPA with incident diabetes were completely attenuated after adjustments for fasting glucose [111]. Mendelian randomization analyses also suggested that insulin resistance had a causal effect on tPA levels. In a more recent, larger cross-sectional population-based study using the EpiHealth study from Sweden, 29 proteins were found to be associated with prevalent diabetes mellitus at a false discovery rate of less than 5%. Of these, 14 of the reported protein associations with T2D were novel [112]. However, Mendelian randomization analyses did not find any causal relationship between these proteins and diabetes, suggesting they may be more useful as biomarkers. Yazdanpanah et al. used proteome-wide Mendelian randomization to identify signal regulatory protein γ (SIRPG), interleukin-27 Epstein-Barr virus–induced 3 (IL27.EBI3) and chymotrypsinogen B1 (CTRB1) as potential drug targets for T1D treatment [113]. In a similar vein, the group utilized available proteomics datasets to identify C-type mannose receptor 2 (MRC2), sodium/potassium-transporting ATPase subunit β2 (ATP1B2), spermatogenesis-associated protein 20 (SPATA20), HP, MANSC domain containing 4 (MANSC4) and α1–3-galactosyltransferase (ABO) as causal proteins for T2D [114].

Overall, proteomics studies have not only led to identification of novel protein biomarkers, but given the pivotal role of proteins in disease pathogenesis, advanced the understanding of diabetes. Advancements in protein profiling techniques have allowed for the measurement of a greater number of circulating proteins with higher specificity. These developments have the potential to improve our understanding of diabetes and other diseases, leading to better diagnosis and potential novel treatment options in the future.

## Application of Metabolomics in Diabetes

Metabolomics, an expanding field of scientific research, has its roots in early metabolite analysis and is now primarily used to identify disease biomarkers, including in diabetes [115, 116]. Diabetes is associated with metabolic disturbances of sugar, protein, fat, water and electrolytes that negatively impact organs such as the liver, skeletal muscle and adipose tissue [117]. Metabolomics offers a comprehensive view, or “snapshot”, of the metabolic landscape, capable of tracking thousands of metabolites across cells, tissues or entire organisms [118, 119•]. The technology can detect the composition of metabolites and their changing trends over time, or after specific perturbations. The combination of metabolic and biochemical information can also highlight the interaction between relevant metabolic and signaling pathways, made possible due to improved analytical techniques and data handling systems [118, 120, 121]. Various platforms for metabolomic profiling are available, such as nuclear magnetic resonance (NMR) spectroscopy, mass spectrometry (MS), Fourier transform infrared (FTIR) spectroscopy and high-performance liquid chromatography (HPLC), and these have all been utilized to advance diabetes research [122, 123].

In the human metabolome, there is a wealth of information regarding low molecular weight metabolites that originate from diet, such as nutrient intermediates, lipids, hormones and other signaling molecules [116]. In a nested case–control study of 503 baseline plasma samples from the Swedish prospective Västerbotten Intervention Programme cohort, taken at a median time of 7 years prior to the diagnosis of diabetes, untargeted liquid chromatography-MS metabolomics led to identification of 46 metabolites associated with incident diabetes, including some novel findings, such as phosphatidycholines (PCs) containing odd-chained lecithins. Many of these metabolites exhibited temporal shifts correlated with the progression toward diabetes, and 42 of the 46 remained significant after adjustments for baseline BMI, fasting glucose and lifestyle factors [124]. Metabolomic studies have also highlighted the potential role of gut microbiota in the development of metabolic diseases. For instance, higher levels of indole propionic acid, produced by intestinal microbes, correlated with improved insulin sensitivity and a lowered risk of T2D onset in the Finnish Diabetes Prevention Study [125]. Large-scale NMR metabolomic profiling has also been conducted in the large China Kadoorie Biobank. The study identified 163 metabolites related to the risk of developing T2D, and 147 of these remained statistically significant after controlling for baseline glucose levels. Elevated levels of specific factors, such as the ratio of apolipoprotein B to apolipoprotein A-1, triglycerides, and the branched-chain amino acids leucine and isoleucine, were all linked to an increased risk of incident T2D [126].

Lipidomics, a specialized subfield of metabolomics, employs high-throughput methodologies to elucidate changes in lipid composition and expression. A study using HPLC-multiple reaction monitoring (MRM) measured 667 serum lipids in subjects with incident diabetes and their matched controls, revealing 38 lipids significantly correlated with T2D risk. These included triacylglycerols (TAGs), lyso-phosphatidyl inositols, phosphatidylcholines, polyunsaturated fatty acid (PUFA)–plasmalogen phosphatidylethanolamines (PUFA-PEps) and cholesteryl esters. This lipidomic profile enhanced predictive accuracy beyond traditional clinical risk factors [127].

In summary, metabolomics represents a powerful tool for identifying metabolic disturbances in diabetes and potential biomarkers for early diagnosis and targeted therapy [103]. As the technology continues to advance, it has the potential to considerably expand our understanding of the pathogenesis of diabetes and pave the way for precision medicine.

## Integrating Omics Research for Novel Discoveries and Precision Medicine

**Fig. 1:**
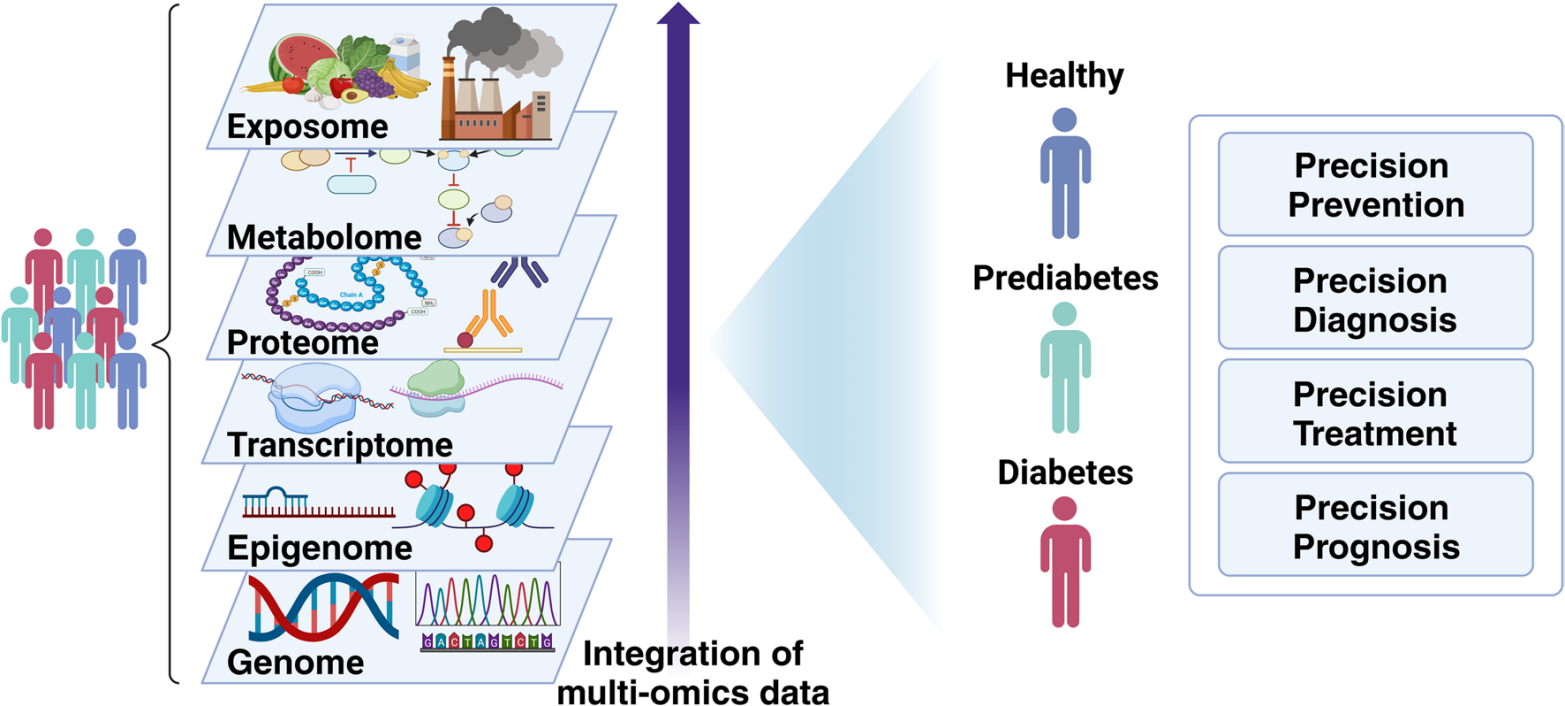
The integration of multi-omics analyses in individuals from cohorts can help to drive the development of precision medicine in diabetes. Legend: each layer represents increasing complexity which have arisen from the genome, epigenome, transcriptome, proteome, metabolome and exposome. These information, representing deep phenotyping of individuals, may provide information that can help inform and guide disease classification and treatment selection, as well as predict future risk of complications. Precision Medicine in Diabetes reflects the overall efforts to utilize these as well as clinical information to guide treatment selection and clinical decisions

Whilst the earlier sections have highlighted recent advances to use epidemiological cohorts to identify different biomarkers for diabetes and related complications, it is the integrative analysis of multiple omics that are likely to provide the most useful novel insights towards diabetes and related complications. In addition to the integration of different layers of omics datasets from different cohorts, there should be considerable advantage in leveraging the measurement of multiple omics in the same individuals (Fig. 1). The UK Biobank, in which the large-scale collection of detailed information, prospective follow-up, whole genome genotyping and exome sequencing, proteomics and metabolomics, and the availability of access to the data for *bona fide* researchers, has been transformative in its global impact on biomedical research [128, 129]. Earlier multi-omics studies have utilized the genetic data to integrate the proteomics data for protein quantitative trait loci (pQTL) analyses to identify genetic variants that regulate protein expression and provide genetic instruments to explore causality between protein biomarkers and different diseases using the MR framework [112], and likewise for methylation quantitative trait loci (meQTL) analyses for methylation markers [88]. In addition, multi-omics data has been generated in an increasing number of cohorts, including the INTERVAL study [110, 130], the Fenland cohort [131], China Kadoorie Biobank [126], FinnDiane [132] and other cohorts. Similar work is ongoing in the Hong Kong Diabetes Register, Hong Kong Diabetes Biobank and the TRansomics ANalysis of Complications and ENdpoints in Diabetes (TRANSCEND) Consortium [93, 133–135]. This generation of deep phenotyping with multi-omics data in large epidemiological cohorts, preferably with prospective follow-up, represents another important dimension in “big data” analytics. The integration of whole exome sequencing data with metabolomics data has facilitated the identification of novel associations implicating rare coding variants, and can advance future drug development [136]. Based on first principle, genome is the most upstream causal factor (the concept underlying MR) with epigenomes, proteome and metabolome, being regulators and mediators expressed as phenomes further modified by age, sex, exposome, demographic microbiome and intervention including pharmacological and non-pharmacological. Whilst the discovery of biomarkers can be epidemiology-focused, unravelling the meaning and applications is very much a clinical science, further complicated by the importance of health beliefs and behaviour for a chronic disease such as diabetes. There are also numerous successful examples where the incorporation of omics research into clinical trials have helped to identify novel biomarkers for clinical outcomes as well as biomarkers related to treatment response [137–141], though there are different limitations including the sample sizes of clinical trials being powered to detect differences in the primary outcomes in relation to the interventions being examined, and relatively short duration of the intervention. Given the complexity of the subject and the inter-disciplinary nature of this kind of work, there are significant advantages for such work to be led by physician researchers, epidemiologists or researchers with knowledge in epidemiology, human biology and medical treatment, in close collaboration with and supported by allied health professionals (e.g. diet, exercise, behavioural), molecular biologists, geneticists, data scientists and members with complementary expertise (e.g. chemistry and drug development) to tackle this complex subject and advance the field.

In this review, we have provided some representative examples of omic studies as applied to the epidemiological investigation of diabetes and related outcomes. It is only possible to provide a snapshot of the most commonly employed technologies, and there are numerous other omics platforms that we did not have space to discuss in more detail, in particular radiomics or imaging data, as well as gut microbiome and exposome [142, 143]. For investigators planning to embark on epidemiological analyses with biobanking for future omics analyses, it is important to be forward-looking, and not be limited by currently available technologies. Other challenges investigators are likely to encounter in this area of work include the capacity and costs for data storage, capabilities for data linkage, methodologies for data analysis, integration of data across the different omics platforms and clinical phenotypes, and the need for multidisciplinary collaboration given the different domains of expertise required. Whilst the different omics are tools with the potential utility to elucidate disease mechanisms, classify disease subtypes, stratify risk and inform targeted treatment, when analyzed in an integrated manner, may provide new insights for predicting, preventing, classifying and personalizing care in diabetes. Advances in the development of methods, including those involving machine learning and artificial intelligence, to integrate data from these multi-omics datasets, would also be key to future development in this area for precision medicine in diabetes [144, 145, 146••].

## Data Availability

Not applicable.
